# Supplementary material for: Are carbon emissions trading and green financial instruments synergistic? -Comprehensive quantitative research based on content analysis
Source: PLoS One. 2024 Mar 7;19(3):e0298601. doi: 10.1371/journal.pone.0298601 (PMC10919601; doi:10.1371/journal.pone.0298601)
Supplement: S1 Data — (ZIP) [file pone.0298601.s001.zip › Data/地级市Z_F工作报告.zip!/░▓╗╒/┴∙░▓╩╨/└┤╫╘╣1⁄2╓┌║┼í╛┬φ┐╦╩2╛▌═°í┐_2013.docx]

各位代表：

　　现在，我代表市人民政府，向大会报告工作，请予审议，并请市政协委员和其他列席人员提出意见。
　　一、团结拼搏，稳步前进的2012年
　　刚刚过去的2012年，全市人民在市委的坚强领导下，牢牢把握稳中求进的工作总基调，抢抓机遇，团结拼搏，基本完成了市三届人大三次会议确定的年度目标任务。预计全市生产总值910亿元，增长11.2%。实现财政收入112.4亿元，增长20.2%。

　　一年来，我们积极应对经济下行严峻形势，全力促进经济平稳增长。及时出台了稳增长的一系列政策措施，有效促进了经济企稳回升。预计实现规模工业增加值350亿元，增长17.8%；新增规模企业98家，总数达到770家，其中亿元企业315家。狠抓项目工作，完成全社会固定资产投资680亿元，增长22%，开发矿业、应流硅溶胶、迎驾曲酒、君诚环保塑业、天通电子、艾莱依服饰、丽人木业等项目建成投产。积极与央企、知名民企对接，与华润集团、中国电子科技集团38所、保利集团签订战略合作框架协议，华润啤酒∕水饮品双百万吨等项目顺利开工，博微长安科技园、万佛湖旅游等项目建设扎实推进。精心组团参加中博会、厦洽会等活动，成功承办第三届中国国际羽绒节，预计全年招商到位内资690亿元、外资2.99亿美元，分别增长26.3%、38.8%。加强政银企对接，预计全年新增贷款突破百亿元，增幅超过17%。努力扩大消费需求，预计实现社会消费品零售总额383亿元，增长15.5%。海关、商检在六安设立了办事机构，预计实现进出口7.2亿美元，增长15%，新增进出口实绩企业40家。

　　一年来，我们着力巩固农业基础地位，大力改善农村生产生活条件。实现粮食总产89.8亿斤，增长4.8%，连续九年创历史新高，获全省粮食生产“三大行动”先进市称号。大力发展现代农业，新增省级现代农业示范区1个、省级农业标准化示范区2个，新增省级龙头企业27家、市级83家。深入推进农业产业化倍增计划，预计实现加工业产值600亿元，增长20%。全面实施百万亩油茶产业发展行动计划和茶产业振兴工程，全国油茶产业发展现场会在我市召开。加强农村基础设施建设，完成农田水利建设投资21亿元，新增灌溉面积24万亩，治理水土流失面积112平方公里，新实施8条中小河流治理和166座小水库除险加固，解决了46万人饮水安全问题。全面启动美好乡村建设，实施农村清洁工程34个乡镇，完成村庄整治23个，改造农村危房14416户。

　　一年来，我们加快发展方式转变，积极推进结构调整。深入开展技术创新工程试点，新培育省市级创新型企业9家、知识产权优势企业12家、工程技术研究中心13家；全年专利授权1530件，增长39%，其中授权发明专利增幅居全省第2位。大力发展高新技术产业，全年新认定国家高新技术企业27家，新增省级高新技术产品93个，预计实现产值254.6亿元、增加值66.6亿元，分别增长34.7%、31.9%。市生产力促进中心晋升为国家级示范中心。积极培育战略性新兴产业，预计实现产值113亿元、增长40%。扎实开展“质量强市”活动，新增安徽名牌产品18个、安徽著名商标12件，“霍山灵芝”申报国家地理标志保护产品获得受理。扎实开展“营改增”试点工作。全面实施旅游业发展第二个三年行动计划，天堂寨成功争创国家5A级景区，预计全年接待国内外游客1500万人次，实现总收入95亿元，分别增长53%、45%。加强节能减排，启动实施节能技术改造项目30个、污染减排项目84个，新建污水管网120公里。积极推进生态市建设，全年创建国家级生态镇3个、生态村2个，省级生态乡镇6个、生态村6个。启动实施“千万亩森林增长工程”。

　　一年来，我们抢抓重大发展机遇，扎实推进区域合作。贯彻落实吴邦国委员长视察六安重要讲话精神，启动实施金寨 “5+1”项目，各项工作进展顺利。深入贯彻中央和全省扶贫开发会议精神，编制《六安市扶贫攻坚规划（2011—2020年）》，出台《关于进一步促进扶贫开发工作的若干意见》。加快开发园区建设，13个省级开发区新增建成区面积20平方公里，积极推进六安开发区创建国家级开发区。市集中示范园区完成基础设施投入4.3亿元，累计开工项目10个，胜昔新材料项目建成投产。加快融入合肥经济圈，加强与合肥市在基础设施规划、旅游推介、联合招商、人才培养等方面的对接合作，启动寿县蜀山现代产业园起步区建设，完成合六产业走廊规划编制，开工建设312国道六安段升级改造、炎刘至新桥国际机场快速通道等项目。

　　一年来，我们持续加快城镇化进程，不断提升承载能力。坚持规划引领，开展城市发展战略研究和东部产业新城建设规划、产业发展规划编制工作。完善城市重点工程建设管理体制，组建市重点工程建设管理局，强化规划及土地集中统一管理。深化投融资体制改革，加强政府融资平台建设，市城投公司多渠道融资28.3亿元，清收历年土地出让金12.6亿元。实施凤凰河、大雁河综合治理改造和淠河城北橡胶坝、西部生态新区路网、亚艇赛场馆建设等7大类56项政府投资项目，完成投资26亿元。提升城市管理精细化水平，开展小街巷环境综合整治，完成城区公厕改造并免费开放。巩固和扩大“国家园林城市”创建成果，启动“五城同创”工作。开展新一轮县城总规修编。加强交通基础设施建设，开工建设大别山旅游扶贫快速通道，实施县乡道改造633公里、危桥改造89座。

　　一年来，我们着力推进文化繁荣，全面发展各项社会事业。实施文化强市战略，出台《关于进一步推进文化强市建设的实施意见》。深入践行社会主义核心价值体系，启动省级文明城市创建活动。文化产业发展取得新进展，悠然蓝溪、新桥国际阳光半岛文化产业园等项目加快推进，预计实现文化产业增加值45亿元，增长20%。妥善解决文艺院团改制等遗留问题，安广网络六安分公司、六安广电网络中心成立运转，六安广播电视报社完成转企改制，再次荣获“全国文化体制改革先进地区”称号。实施文化惠民工程，新建成9个乡镇综合文化站、93个公共电子阅览室、238个农家书屋，广播、电视综合人口覆盖率达95%和92.2%。成功举办首届中国大别山（六安）山水文化旅游节。基层医改不断深化，县级公立医院综合改革全面启动，城乡医疗救助“一站式”管理服务网络实现全覆盖。教育改革发展步伐加快，启动实施六安一中东校区建设，霍山职高、霍邱陈埠职高通过国家级改革示范校评估。广泛开展群众性体育活动，成功举办市三运会和市第九届老运会。切实加强流动人口管理和出生人口性别比治理，低生育水平保持稳定，人口自然增长率为7.1‰。继续加强社区建设，基础设施条件进一步改善。国防动员和民兵预备役工作扎实推进，国防教育和双拥共建取得新成效。统计、人防、档案、民族宗教、外事侨务、对台、气象、地方志、防震减灾、妇女儿童、新闻出版、老龄、残疾人等各项事业取得新进展。

　　一年来，我们大力保障和改善民生，切实维护社会和谐稳定。强力实施33项民生工程，累计投入财政资金60亿元，惠及700万城乡居民。大力实施“十二五”城乡居民收入倍增规划，预计城镇居民人均可支配收入19403元，增长13.5%；农民人均纯收入6489元，增长15%。成功发行“城市一卡通”。新农合实现应保尽保。城乡居民养老保险实现全覆盖，基础养老金发放率100%。城乡最低生活保障实现应保尽保，累计发放低保资金5.86亿元。新改扩建农村敬老院42所，五保供养标准大幅提高，集中供养率达32%以上。开工建设保障性安居工程25218套，基本建成20414套，发放廉租房补贴12597户，超额完成省下达任务。做好就业再就业工作，城镇新增就业5.73万人，下岗失业人员再就业2.33万人，城镇登记失业率控制在3.76%以内。加大扶贫工作力度，争取财政扶贫资金1.57亿元，整村推进196个。切实加强新形势下群众工作，创新基层社会管理和服务体系，社区网格化管理覆盖率达47.2%；强化特殊人群监管安全和弱势群体法律援助，积极构建社会矛盾大调解体系，深入开展领导干部开门接访、带案下访和包案处访活动。持续加大安全生产和食品药品安全监管力度，严厉打击刑事犯罪，人民群众安全感不断提升。

　　一年来，我们高度重视政府自身建设，进一步提高行政效能。扎实开展“保持党的纯洁性、迎接党的十八大”主题实践活动。坚持科学决策、民主决策，健全重大行政决策风险评估、专家论证、合法性审查和集体讨论决定制度。深入开展政务公开政务服务“标准化建设年”活动，深化行政审批制度改革，全年清理规范性文件678件。自觉接受人大监督和政协民主监督，进一步密切与工会、共青团、妇联等人民团体的联系。认真办理《关于逐步提高五保集中供养、机构建设和管理费用标准的议案》等人大代表议案和建议137件，办理《关于加快发展公益性幼儿园的建议》等政协提案228件，办复率100%。积极回应网络问政，办理省长留言36批次81件，回复网民留言13474条。突出抓好廉政风险防控，强化对重点领域和关键环节的行政督察和审计监督，勤政廉政建设取得新成效。

各位代表！过去的一年，六安在转型中发展，在逆势中前行，势头积极向好，成绩来之不易。这是省委、省政府和市委正确领导的结果，是全市人民同心同德、奋力拼搏的结果。在此，我代表市人民政府，向全市各界人士表示衷心的感谢!

　　在看到成绩的同时，更要清醒地认识到，在全省我市经济总量居中、人均居后的格局没有改变，发展不快、发展不优的现状没有改变，一些矛盾和问题尤为突出，主要是：投资、消费增长乏力，经济下行压力加大，缺少大企业、大项目支撑；产业结构不尽合理，主导产业没有真正形成，第三产业发展滞后；土地、资金等要素供需矛盾仍然突出，节能减排任务艰巨；基本公共服务体系还不健全，社会建设和改善民生还有大量工作要做。政府自身建设还不完全适应形势发展需要，少数部门和干部作风不实、责任心不强，执行力、落实力有待进一步增强。对此，我们将高度重视，采取更加切实有效的措施加以解决。

　　二、攻坚克难，扎实做好2013年工作
　　今年政府工作的总体要求是：全面贯彻党的十八大精神，以科学发展为主题，紧紧围绕“五位一体”总体布局，牢牢把握“四化同步”战略部署，坚持稳中求进、好中求快，致力提速增效、争先进位，深入实施招商引资主战略、工业化核心战略和城镇化带动战略，更加注重平稳较快增长，更加注重发展方式转变，更加注重民计民生改善，更加注重生态文明建设，更加注重社会和谐稳定，全力加快幸福六安建设。

　　综合考虑，今年主要预期目标是：生产总值增长12%，财政收入增长15%，固定资产投资增长22%，规模工业增加值增长16%，社会消费品零售总额增长15%，城镇居民人均可支配收入增长14%，农民人均纯收入增长15%，城镇登记失业率控制在4.2%以内，人口自然增长率控制在7.2‰以内，完成年度节能减排目标任务。

　　实现上述目标任务，必须着力抓好以下十个方面工作：

　　（一）全力保持经济持续健康较快发展

　　提升投资和项目工作水平。启动实施重点项目建设“253工程”，争取更多项目进入省“861”行动计划。加快首矿大昌铁矿深加工、华电二期、华润啤酒∕水饮品双百万吨、江淮电机产业园、圣农石斛产业园、创凯电子等一批重点项目建设。推进亚夏汽车财富广场、博微长安科技园、应流特种高端装备制造、金日盛矿业云母深加工、索伊电器二期、武钢产业园、金寨钼矿开发以及寿西湖行洪区调整、济祁高速六安段、合六南通道等国省干道建设早日开工。加强项目谋划，抢抓中央政策机遇，围绕保障性安居工程、“三农”、城市基础设施、民生工程、节能减排、生态建设等领域，积极谋划、编报一批重大项目，提升项目前期工作效率和水平。积极拓宽民间投资领域和范围，鼓励和引导民间投资。进一步加大与央企、知名民企对接力度，加速签约项目转化实施。

　　强力推进招商引资。持续加大招商力度，切实提高招商实效，确保引进内资和外商直接投资均增长15%以上。突出产业招商，编制完善市产业招商指导目录，围绕主导产业、核心企业和产业链上下游配套，开展精准招商，加速产业集聚。推进与发达地区商会、协会、开发园区、企业、科研院所的合作，深化以商招商。健全招商机制，整合市直8个产业招商小组与驻外联络处力量，完善项目会商推进机制和跟踪服务机制，建立项目准入审查、产业布局协调机制。强化部门协调联动，统筹重大项目布局和招商优惠政策，推进县区错位协调发展。抓好中国光彩事业大别山革命老区行活动。办好第四届中国国际羽绒节。

　　大力促进消费和进出口。落实促进消费的各项政策措施，完善城乡流通体系，大力开拓农村市场，积极引导农民消费。继续推进“五位一体”便民商贸服务体系建设，培育和引进大型商贸企业，优化空间布局，改善消费环境。积极发展电子商务和会展经济。保持房地产业持续健康较快发展，引导合理需求，规范市场秩序。着力扩大进口和加工贸易规模，开拓服务贸易、小额旅游购物等新兴对外贸易方式，促进外贸均衡发展，力争进出口总额增长15%以上。争取设立出口加工区、保税仓库。

　　（二）进一步突出工业化

　　培育产业优势。加快实施重点产业规划，深入推进千亿产业培育工程和百亿产业培育计划，力争9大主导产业增加值占全部工业增加值82%以上，实现钢铁相关产业产值260亿元。针对产业链关键环节，通过产业招商和骨干企业结构调整，实施百项以上产业配套项目。突出各县区产业发展重点，依托各省级开发区打造10个左右特色优势产业基地。

　　壮大骨干企业。坚持扶优扶强、一企一策，支持迎驾集团、应流集团、大昌矿业、索伊电器、金安不锈钢、龙华集团等企业通过扩建改造、联合重组、股改上市等方式快速扩张。选择产品技术含量较高、发展基础较好的百户成长型企业，组织实施50项以上投资额超5000万元的技术改造及技术创新项目，加快推动工业化与信息化融合发展，力争全年新增亿元企业30家。

　　扶持中小企业发展。认真落实促进中小企业发展的政策措施，加快中小企业公共服务平台、信息服务网络和创业基地建设，健全中小企业服务体系。进一步完善支持政策，优化发展环境，积极推进全民创业，鼓励社会资本投入实体经济。做大工业投资和中小企业融资担保平台，创新运作模式，切实缓解中小企业融资难问题。

　　（三）加快新型城镇化

　　加强城乡规划编制与管理。健全城乡规划体系，完善中心城区控制性规划，完成东部产业新城、西部生态新区总体规划、控制性详细规划编制，加快相关专项规划编制进度；完成五县县城、叶集城区总体规划修编及霍邱钢城、新桥空港新城总体规划编制。围绕滨水、绿色、人文特色，加强中心城市重点区域、重要地块的修建性详规编制。按照统分结合的原则，完善城乡规划管理体制。

　　全方位、大力度推进中心城市建设。深入开展“五城同创”和国家森林城市创建工作。全力推进第二轮中心城市建设重点工程三年实施计划，投资40亿元左右，重点实施好312国道六安段升级改造二期、六安火车站扩建、淠河两岸防洪治理、解放路综合交通建设等项目，着力完善配套、提升功能。强化工作推进机制，坚持责权利统一，落实重心下移，强化协调调度，形成工作合力。强力推进征迁工作，理顺工作机制，坚持市区联动，完善相关政策，维护群众合法利益。切实加强招投标管理，规范招投标行为，实行决策、监督、执行相分离，加强标前、标中、标后监管，创造公平竞争的市场环境。立足新起点，瞄准新目标，进一步提升城市管理精细化、规范化水平。

　　分类推进县城和集镇建设。加快金寨新县城、霍邱钢城、寿县新城区、叶集北部新城建设步伐，支持霍山县城争创国家园林县城，推进舒城、霍邱、寿县等有条件的县城规划建设中等城市。进一步加快各县城镇基础设施和公共服务配套设施建设，着力扩容提质、完善功能，增强承载能力。继续推进扩权强镇试点。突出50个重点镇建设，打造一批特色鲜明的工业强镇、商贸重镇和旅游名镇，有序引导人口、产业向城镇集中。坚持城镇基础设施和公共服务设施向农村延伸，加快城乡一体化进程。

　　（四）推进农业现代化

　　提升农业综合生产能力。以科技创新引领农业转型升级，扎实推进粮食生产“三大行动”，确保粮食产能稳定在百亿斤以上。深入实施畜牧业升级计划、水产跨越工程以及“菜篮子”工程，集中创建一批省部级标准化养殖示范场、蔬菜标准园。大力发展品牌农业，推行标准化生产，力争无公害、绿色和有机“三品”认证总数达到230个。全面实施农村水利综合治理，积极争取淠河、史河防洪治理项目，实施10条中小河流治理，完成90座病险小型水库除险加固，治理水土流失面积80平方公里。

　　加快农业产业化步伐。深入实施农产品加工“168”工程，力争全年农产品加工产值突破700亿元，增长12%以上。大力实施百万亩油茶产业发展行动计划和茶产业振兴工程，加快金安、金寨现代农业示范园及霍邱长集农产品加工园等一批省级农业产业化示范园区建设，积极培育粮食油脂、畜禽水产、酒茶果蔬等一批百亿元产业集群，支持毛竹、石斛、白鹅等特色农产品基地建设。坚持培育与引进并重，做大做强龙头企业，着力把农产品资源优势转化为产业优势。

　　创新农业社会化服务体系建设。健全农业科技服务和管理机制，深入推进专家大院建设和科技特派员创业行动。加大政策性农业保险实施力度，提高农业风险保障水平。创新农业经营体制，激活农村土地流转市场，完善流转服务体系，推进土地规范、有序、高效流转。加快供销合作社改革发展，健全农资销售服务体系。大力发展农民合作组织，鼓励农民开展资金互助、土地股份和农村社区合作。

　　全力抓好美好乡村建设。贯彻落实《关于全面推进美好乡村建设的实施意见》，重点实施首批231个中心村和600个自然村建设，全面推进规划编制、基础设施建设、特色主导产业培育、环境绿化美化、公共服务配套、社区化管理等重点工作，引导农民向中心村集聚。统筹安排和整合涉农项目资金，集中投入中心村建设。建立结对共建机制，鼓励社会资本参与美好乡村建设。

　　（五）加快转型发展

　　提高自主创新能力。加快科技企业孵化平台、农业科技创新平台和生产力促进中心建设。深化产学研结合，认真落实与合工大、安农大战略合作协议。加速科技成果转化应用，新培育创新型企业6家、工程技术研究中心8家、知识产权优势企业8家。加快发展高新技术产业，全年新培育国家高新技术企业20家以上、省高新技术产品100个以上。加强专利创造、运用和保护，实现申请专利3000件以上，授权专利1800件以上。推进商标品牌战略，加快六安国家级茶及茶制品质检中心、应流国家级企业技术中心、舒城省级童车检测中心建设。

　　加快发展战略性新兴产业。充分发挥财政专项资金的引导作用，组织实施战略性新兴产业“倍增工程”。围绕新能源、节能环保、新材料、生物、电子信息、高端装备制造等领域，建立战略性新兴产业项目库，引进和新上一批重点项目，争取国家和省重大项目在我市布局。支持蓝博旺工程车辆、凯迪循环经济园、江淮电机、圣农生物、沃巴弗电子、振华光电等企业做大做强。

　　重视发展现代服务业。制定服务业发展规划，重点培育科技研发、金融保险、物流配送、中介服务等生产性服务业，大力发展商业连锁、中高档酒店、休闲娱乐、社区服务等生活性服务业。加快吉宝皖江国际冷链物流园、托马斯物流中心、新加坡新桥国际物流园等现代物流园区建设。打响大别山旅游品牌，加快六安中心城区旅游集散中心建设和以天堂寨、白马尖、万佛湖、寿县古城、六安中心城区及城郊为中心的五大旅游区建设。大力发展红色旅游和乡村旅游。完善旅游基础设施，强化重大项目带动，整合旅游资源，推动旅游与文化、科技融合发展。加快悠然蓝溪、大别山玉石等一批文化产业园区建设，重点支持皖西演艺集团等文化企业发展，着力提升文化产业规模化、集约化和专业化水平。

　　推进生态建设。建立重大生态修复工程项目库，积极争取建立大别山区生态补偿机制。加强水源地保护和水土保持，完成库区饮用水源保护和周边环境质量提升工程三年行动计划，推进瓦埠湖综合治理，编制实施淠河国家湿地公园建设规划。强力推进“千万亩森林增长工程”，切实加强野生动植物保护和重大有害生物防治。开展循环经济试点，培育节能环保产业。扎实推进节能减排，加快淘汰落后产能，深入开展万千家企业节能低碳行动。围绕“十二五”水污染防治目标要求，加快凤凰桥、城南等污水处理厂及配套管网建设，加强规模化畜禽养殖场污染防治，深入开展生态创建和农村环境连片整治。

　　（六）推动区域协调发展

　　全面实施新一轮扶贫攻坚。创新扶贫开发机制，大力推进行业扶贫、专项扶贫和社会扶贫。紧紧抓住大别山片区区域发展与扶贫攻坚规划启动实施的重大机遇，做好项目谋划，积极争取国家贫困地区发展基金。加强与中直、省直对口帮扶单位的联络与协调，争取更多更大支持。继续落实扶贫开发“一把手”负责制，扎实做好领导联系帮扶和部门对口帮扶贫困村工作。积极开展贫困家庭子女教育资助活动，提升贫困群众综合素质，增强贫困家庭的造血功能。

　　强力推进“5+1”项目。以 “5＋1”项目为抓手，充分发挥其示范引领、辐射带动作用，着力在“抓金寨、带全市”上取得成效。加快现代产业园起步区建设，全方位、大力度开展招商引资，争取引进一批央企、知名民企入驻。高标准推进职业学校建设，确保按时建成招生。加快农村金融综合改革，有效提升县域金融服务能力。积极筹措资金，加快大别山旅游扶贫快速通道建设。加快金寨抽水蓄能电站前期工作进度，确保年底前申报国家发改委核准。

　　全方位开展区域合作。深入实施皖江示范区规划，围绕东部产业新城、合六工业走廊、舒三工业集聚区和新桥国际产业园产业布局，打造产业承接主平台，提高产业承接水平。加快落实合肥经济圈城市党政领导会商会议成果，深化寿县、霍邱与合肥市的结对合作，加快寿县蜀山现代产业园、霍邱职业教育园建设，加快推进合肥—新桥国际机场—六安城际轨道交通项目前期工作，合作开通合肥—新桥国际机场—新桥国际产业园城市公交专线。

　　（七）提升开发园区发展水平

　　完善园区规划。按照产城一体的要求，统筹全市开发园区发展。完善各省级开发区总体规划、产业发展规划和相关专业规划，明确功能定位，优化空间布局，彰显各自特色，实现错位发展。推进各省级开发区以“一区多园”、“托管区”、合作共建等形式整合周边乡镇工业集中区。全力争取市开发区升级为国家级开发区。

　　提升承载能力。高标准推进园区基础设施建设，完善功能配套。依托园区现有条件，加快建设中小企业创业园，支持初创企业；科学规划并加快建设标准化厂房，满足中小企业快速入驻。创新园区开发模式，加强与央企、知名民企、高校等合作共建，吸引域外资金投资发展“飞地经济”。

　　突出主导产业。制定出台全市园区产业发展指导目录，省级开发区重点培育2-3个主导产业，其他园区重点培育1-2个主导产业，突出首位产业，打造特色产业。围绕主导产业开展招商，延伸产业链，促进产业集聚。坚持自主创新和技术引进相结合，大力发展高新技术产业，推进传统优势产业与新兴产业融合发展，引导园区产业升级。

　　创新体制机制。完善东部产业新城管理体制，形成开发建设合力。进一步理顺各园区与所在行政区的关系，在土地、规划、建设、财政及内部管理等方面赋予园区更多自主权。坚持精简、效能原则，创新园区运行机制，大力推行扁平化管理。加强园区服务功能建设，提升服务效能。

　　推进节约集约发展。按照产城融合的要求，完善开发园区城市功能。加强园区与城区基础设施对接，实现一体化布局和联动发展。严格执行产业规划、环境保护、投资强度、税收贡献等项目准入标准，推进园区可持续发展。加强园区土地集约利用，鼓励新上项目建设多层厂房，支持现有企业厂房加层改造以及实施内涵式技术改造。

　　（八）致力发展要素保障

　　推进金融创新。深入开展金融创新年活动，扎实推进政银企合作，引导金融机构加大有效信贷投放，确保全市贷款增量所占全省份额持续增长。推进农村金融改革，完成市农村商业银行组建，启动霍邱、寿县信用联社改革，推动县级农合行向农商行转制。进一步拓宽融资渠道，加快引进域外金融机构和异地资金，推进市级金融网点向县乡延伸。加快应流机电、迎驾贡酒、龙华竹业等企业上市融资，支持企业发行融资债券，发展创业投资和股权投资。设立中小企业集合票据发行偿债基金、风险代偿基金和金融机构贷款奖励基金，促进社会融资规模结构加速优化。加强金融生态建设，维护金融稳定。

　　做大做强融资平台。进一步明晰市城投公司等融资平台的职能定位，完善法人治理结构，做大资产规模，拓展投融资渠道，创新投融资模式，增强投融资能力。强化经营城市理念，坚持政府主导、市场运作，引导更多社会资金投入城市基础设施。完善投融资管理体制。力争发行第三期城投债，抓好社保基金、住房公积金委托贷款用于保障房建设试点工作。

　　强化土地资源管理。严格耕地红线保护，优化配置土地资源。用足用活土地管理政策，通过独立选址、未利用地、增减挂钩、各类奖励等途径积极争取土地指标。加强闲置土地、低效用地处置，盘活存量土地。加强市区建设用地集中管理，统一土地储备、供应、登记和执法。加大土地执法监察力度，确保卫片执法检查“零问责”和土地例行督察顺利通过。完善土地收购、储备机制，加快土地要素市场建设，强化土地出让金清缴和征收管理。

　　深化重点领域和关键环节改革。深化行政审批制度改革，推进部门内部职能整合和业务流程再造，完善基本建设项目联合审批平台。积极稳妥推进户籍管理制度改革，有序推进农业人口转移。进一步规范税收征管秩序，完善市区财政体制。推进全口径预算管理，深化预算制度改革，强化预算监督。落实结构性减税政策，深入开展 “营改增”试点。全面开展县级公立医院综合改革，深化基层医疗卫生综合改革。积极稳妥推进事业单位分类改革。全面加强国有及国有控股企业监督管理，实现行政事业单位国有资产集中管理和统一运营，确保国有资产保值增值。加强和改进政府投资审计工作。

　　（九）切实保障和改善民生

　　巩固提升民生工程。按照省政府要求，实施33项民生工程，落实配套资金，加强资金和项目管理。健全工程运营管理机制，注重后期管理养护。按照量力而行、尽力而为的原则，认真解决好广大人民群众普遍关注、迫切需要解决的突出问题。积极推进市民居家养老服务体系建设，加强城市小街巷综合治理，改善城市公共交通，扩大“城市一卡通”覆盖范围。

　　多渠道增加就业。进一步完善覆盖城乡的公共就业服务体系和人才服务体系，力争全年城镇新增就业3.1万人，下岗失业人员再就业1.5万人。改进小额贷款发放，支持下岗失业人员再就业。继续对困难高校毕业生实行就业帮扶，推进农村劳动力转移就业，加强对城镇就业困难群体的就业援助。开展“充分就业社区”创建活动，全面落实就业培训和岗位补贴等优惠政策。

　　完善社会保障体系。进一步扩大社会保险覆盖面，提高统筹层次，巩固城镇职工养老保险省级统筹成果，完善失业、医疗、工伤保险市级统筹。大力推进城乡居民养老保险，力争参保370万人。健全完善新农合制度，积极推进大病保险制度和支付制度改革，稳步提高保障水平。健全物价调控监管长效机制，完善价格补贴联动机制。大力推进保障性住房建设、分配和管理，完善普通商品房供给，努力满足居民多层次住房需求。

　　增加城乡居民收入。稳步推进“十二五”城乡居民收入倍增规划，拓宽居民投资渠道。认真落实各项强农惠农富民政策，抓好对农业、农民各项政策性补贴的兑现发放，严格执行粮食最低收购价、农产品价格保护等政策措施，发挥政策性增收效应。加大对低收入群体、特殊困难群体社会救助力度，增加居民转移性收入。鼓励引导劳动力就近就业创业，提高居民工资性收入。加强再分配调节，完善最低工资制度和工资集体协商制度，严格执行最低工资标准和农民工工资支付保障制度。

　　（十）全面加强和谐社会建设

　　加快文化强市建设。认真贯彻落实《文化强省建设实施纲要》，制定推进文化强市建设三年行动计划。组织实施公民素质提升工程，大力弘扬新时期六安精神，广泛开展群众性精神文明创建活动。深入挖掘文化资源，加快文艺创作，力争推出一批具有六安地域特色的精品力作。加强非物质文化遗产抢救与保护，支持寿县古城保护与开发。继续组织中国大别山（六安）山水文化旅游节、大别山歌会等活动。积极发展社区便民文化，抓紧论证、完善市图书馆、档案馆、科技馆、文化馆和新皖西大戏院建设方案，及时开工建设。

　　统筹发展社会事业。继续推进学前教育三年行动计划和义务教育阶段学校标准化建设，新建、改扩建一批中小学校和公办幼儿园。探索高中招生制度改革，促进普通高中多样化发展。继续加强职业与成人教育，支持高职中职教育集团化发展。健全城乡医疗卫生服务体系，发展民营医疗机构，加快发展社区卫生，认真组织实施重大传染病医疗救治。深入开展爱国卫生运动，全力推进省级卫生城市创建工作。广泛开展全民健身运动，办好2013年亚洲赛艇锦标赛。继续巩固出生人口性别比“重点治理年”成果，稳定低生育水平；创新城市和流动人口计生服务管理，推进诚信计生和利益导向体系建设，促进人口计生工作转型发展。加强统计制度方法改革创新，深入推进国民经济核算和企业“一套表”制度改革，组织开展第三次全国经济普查。依法管理宗教事务，加快民族乡村经济社会发展。加强国防动员、民兵预备役工作，深化巩固双拥工作和国防教育成果，积极支持驻军建设。深入实施妇女儿童发展十年规划，加快养老服务四级网络和残疾人服务体系建设，协调发展工商、保险、邮政、气象、人防、地震、档案、地方志、对台、外事侨务等工作。

　　加强和创新社会管理。坚持以人为本、服务为先，加快形成源头治理、动态管理、应急处置相结合的社会管理机制。健全完善突发性事件预防处置机制，加快构建政府主导、社会力量参与化解社会矛盾的平台。创新信访工作体制机制，进一步畅通信访渠道。加强流动人口、特殊人群以及非公有制经济组织、社会组织和虚拟社会的服务管理，推进刑释解教和社区矫正人员过渡性安置帮教基地建设。建立完善网络管理和舆论引导机制，着力推动社会管理信息化建设。推进社区、村网格化管理，加快城乡社区标准化建设。

　　推进公共安全体系建设。建立完善食品药品质量追溯机制，健全安全应急体系，加强风险监测评估预警和监管执法。加快市政府应急指挥中心建设，有效整合应急力量和资源。持续深入开展“安全生产年”活动，严格落实企业安全生产责任制，加快市级安全生产技术支撑体系建设。深化平安创建活动，加快推进六安城区以及农村视频防控系统建设，加强治安防范工作，加大对盗窃、赌博、非法集资等社会治安突出问题以及重点地区的整治力度。
　　三、求真务实，提高政府执行力和落实力
　　站在新的起点上，面对各地竞相发展、不进则退的逼人形势，肩负与全国同步建成小康社会的光荣使命，顺应全市人民过上更好生活的强烈愿望，加快发展、富民强市的任务比任何时候都更加艰巨而繁重，我们必须全面把握形势、清醒认识差距、坚定发展信心，始终保持昂扬向上的精神、求真务实的态度、埋头苦干的作风，直面矛盾，敢于担当，朝夕勤勉，勇往直前，以更强的执行力和落实力推动六安发展提速进位。

　　加强学习，提升能力。深入学习贯彻党的十八大精神，准确领会十八大确立的重大理论观点、重大方针政策、重大工作部署，更加自觉地运用十八大精神武装头脑、指导实践、推动工作。注重学习新知识、增强新本领，熟悉掌握现代经济、金融、科技、管理知识，提高贯彻执行政策水平。更加注重研判宏观形势、研究上级政策、把握市情民愿，进一步增强工作的超前性和敏锐性，切实提高驾驭复杂局面、解决复杂问题的能力。

　　解放思想，开拓创新。坚持解放思想先行，勇于革除不利于科学发展的思维定势和行为模式，勇于突破不利于加速崛起的条条框框和陈规陋习。坚持把解放思想融入到实践全过程，与解决具体问题结合起来，哪里有问题就在哪里解放思想，哪里打不开局面就在哪里解放思想，鼓励改革，崇尚创新，宽容失败，充分激发创造活力，努力形成强大合力。

　　规范履职，依法行政。进一步规范行政决策程序，健全行政决策规则，完善公众参与、专家论证、风险评估、合法性审查和集体讨论决定等制度，不断提高行政决策科学化、民主化水平。认真实施“六五”普法规划。大力开展市县乡联动的依法行政示范单位创建活动，全面推行行政执法责任制、评议考核制和过错责任追究制，进一步完善行政层级监督机制，坚决纠正、查处违法和不当行政行为。更加自觉接受人大监督、政协民主监督，主动听取各民主党派、工商联、无党派人士及各人民团体的建议和意见，主动接受人民群众和新闻媒体的监督。

　　转变作风，狠抓落实。严格落实中央八项规定，以身作则，率先垂范。切实精简会议文件，减少迎来送往，清理规范检查、评比、达标活动。坚持说实话、办实事，力戒形式主义。对重大项目实施一名领导、一个专班、一套方案、一抓到底。继续深入开展机关效能建设，加大明查暗访和问责力度，大力整治“庸、懒、散、奢”。加强政府绩效管理，加大对政府部门履行职责的检查、监督与考核力度。推进政府信息公开，加强电子政务建设，充分发挥市长热线、在线访谈、在线回复、政风行风热线等平台作用。继续深入开展“社会评窗口、万人评科长”活动，强化督查督办，严格兑现奖惩。

　　廉洁从政，树立形象。全面落实党风廉政建设责任制和责任追究制，突出抓好各级干部特别是领导干部的廉洁自律。切实加强对权力运行的管理和监督，进一步规范建设工程招投标、国土资源交易、产权交易、政府采购等公共资源交易行为。加强机关行政事务管理，压缩“三公”支出，降低行政成本。强化领导干部经济责任审计，坚决查处违法违纪案件，切实纠正损害群众利益的不正之风。注重运用重大典型案件开展警示教育，警钟长鸣，使政府工作人员始终保持清醒头脑，以清正廉洁的政府形象取信于民。

　　各位代表！党的十八大描绘的前景灿烂辉煌，绘就的蓝图激荡人心。让我们在中共六安市委的坚强领导下，高举中国特色社会主义伟大旗帜，万众一心、同心同德，扎实推进幸福六安建设，朝着全面建成小康社会的新目标奋勇前进！
